# Herbs and natural supplements in the prevention and treatment of delayed-onset muscle soreness

**Published:** 2017

**Authors:** Abbas Meamarbashi

**Affiliations:** *Department of Physical Education and Sports Sciences, University of Mohaghegh Ardabili,Ardabil, Iran*

**Keywords:** Herbs, Natural products, Delayed onset muscle soreness

## Abstract

**Objective:**

Unaccustomed and intense eccentric exercise is a common cause of delayed-onset muscle soreness (DOMS). There are multiple remedies for the treatment of DOMS, but its clinical and laboratory pieces of evidence are scarce. Currently, the treatments proposed for DOMS are numerous and include pharmaceuticals, herbal remedies, stretching, massage, nutritional supplements, and other alternatives. To find a holistic treatment with effective pain relief and minimum side effects, complementary and alternative medicine, including herbal therapies, plays a main role.

**Methods:**

In this review, the existing published studies investigating the efficacy of herbal and natural supplementation therapies for the prevention or treatment of side effects, symptoms, and signs of DOMS are summarized.

**Results:**

Previous studies have documented the efficacy of herbal therapies to treat pain, inflammation, as well as laboratory and clinical side effects of DOMS.

**Conclusion:**

The use of herbs in DOMS seems safer and has lower side effects than pharmacotherapy. However, the potential for side effects and drug interactions should be considered.

## Introduction

Any type of physical activity that causes unaccustomed and high loads on muscles may lead to delayed-onset muscle soreness (DOMS). Amateur and professional athletes are concerned about muscular discomfort and pain phenomena, because they can limit further exercise and training activity (Udani et al., 2009 ▶). However, DOMS can appear after any unaccustomed and eccentric activity in normal people. Exercise-induced muscle soreness is classified into acute and delayed muscle soreness. Acute muscle soreness occurs during the exercise and may continue for about 4 to 6 h. Delayed-onset muscle soreness occurs 8 to 24 h after strenuous exercise and peak of this muscle soreness occurs 24 to 48 h after the exercise. Exhaustive eccentric exercise is usually responsible for DOMS. During an eccentric contraction, the muscles must be active when stretched; therefore, repeated eccentric muscle contractions are responsible for inducing delayed-onset muscle soreness. Eccentric muscular contractions in downhill running, hopping, plyometric exercising, squatting, and the lowering phase of lifting weights can produce DOMS (Connolly et al., 2003 ▶). The main symptoms in DOMS are muscular stiffness, tenderness, and pain during active movements (Fridén et al., 1981 ▶); (Gleeson et al., 1998 ▶). There are many symptoms related to the muscle inflammation and damage such as muscle fiber swelling (Fridén et al., 1988 ▶), elevated serum activities of muscle specific enzymes such as creatine kinase (CK) and lactate dehydrogenase (LDH) (Cleak and Eston, 1992 ▶; Tartibian et al., 2009 ▶), as well as reduced muscle strength (Connolly et al., 2003 ▶) and knee joint range of movement (Saxton et al., 1995 ▶).

**Figure 1:**
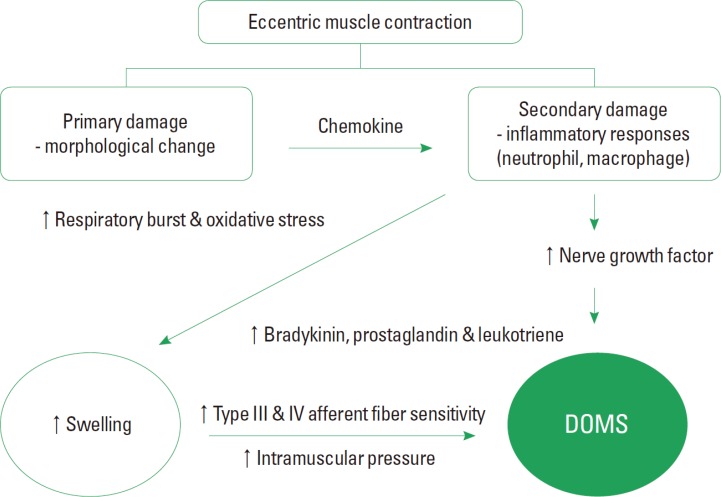
General cytochemical mechanism of DOMS

The mechanisms underlying the cause of DOMS are not fully understood; however, it is generally accepted that DOMS is associated with muscle and/or connective tissue damage and/or subsequent inflammatory responses (Cheung et al., 2003 ▶). Following the muscle injury, enzymatic reactions and inflammatory mediators such as thromboxanes, prostaglandins, and leukotrienes from the cyclooxygenase and lipoxygenase pathways correspond to the increase in vascular permeability and pain perception by sensitizing type III and IV afferent nerve fibers to both chemical and mechanical stimuli (Connolly et al., 2003 ▶; Kim and Lee, 2014 ▶). Swelling results from the movement of immune cells and fluid from the bloodstream into the interstitial spaces with inflammation and can contribute to pain sensation. Increase in vascular permeability causes neutrophils recruitment across endothelium to the site of damage. The muscle microscopic injury is instigated by a mechanical disruption to sarcomeres (Warren et al., 2002 ▶), T-tubules, myofibrils, cytoskeletal protein, and sarcoplasmic reticulum (SR) (Armstrong, 1984 ▶; Child et al., 1998 ▶; Clarkson and Sayers, 1999), which lead to an inflammatory response (Gleeson et al., 1995 ▶). Recent studies have reported that bradykinin and nerve growth factors also play a pivotal role for developing DOMS (Murase et al., 2010 ▶).

Multiple preventive or treatment methods have been advocated to alleviate DOMS. These methods are pharmacological (e.g. non-steroidal anti-inflammatory medications) (Francis and Hoobler, 1987 ▶; Gulick et al., 1996 ▶; Hasson et al., 1990 ▶; High et al., 1989 ▶; Janssen et al., 1983 ▶), exercise (Rodenburg et al., 1994 ▶; Weber et al., 1994 ▶), stretching (Buroker and Schwane, 1989), cryotherapy (Crystal et al., 2013 ▶; Denegar and Perrin, 1992 ▶; Gulick et al., 1996 ▶; Isabell et al., 1992 ▶; Yackzan et al., 1984 ▶), transcutaneous electrical nerve stimulation (Denegar and Perrin, 1992 ▶), ultrasound (Hasson et al., 1990 ▶; Tiidus, 1997 ▶), hyperbaric oxygen therapy (HBOT) and electromagnetic shielding, whey protein (Buckley et al., 2008 ▶), fish oil and isoflavones (Lenn et al., 2002 ▶), caffeine (Maridakis et al., 2007 ▶), l-carnitine (Giamberardino et al., 1996 ▶), antioxidant vitamins (Shafat et al., 2004 ▶), and branched-chain amino acid (BCAA)(Ra et al., 2013 ▶). However, these results are not consistent in attenuating pain or have a small or non-significant effect (Connolly et al., 2003).

In addition, in the last decades, the application of herbs in the prevention and treatment of DOMS is growing. In recent years, herbal supplements have become increasingly popular in the prevention and treatment of somatic pain and discomfort (Arent et al., 2010 ▶; Meamarbashi and Abedini, 2011 ▶; Meamarbashi and Rajabi, 2014 ▶). Interestingly, this application has the advantage of lacking many of the side effects of the common nonsteroidal anti-inflammatory medicines (NSAIDs), such as gastrointestinal distress and cardiovascular complications (Graumlich, 2001 ▶; Mukherjee et al., 2001 ▶). 

There are many different factors which influence the incremental trend of herbal supplementation. Most people believe herbs are cheaper and safer with lower side effects and also are easily available. By contrast, some herbs may have antagonistic interactions with some drugs (Abebe, 2002 ▶). These products are available to consumers as over-the-counter (OTC) and are mostly used to alleviate pain. Most of the medicine used in pain management can be addictive and produce side effects. On the other hand, herbs are mostly safer if being wisely consumed.

**Application of herbs and natural supplements in the prevention and treatment of DOMS**

Some herbs and spices are widely used in the human diet. Traditionally, many benefits of herbs are discovered to alleviate pain and reduce inflammation, which are studied or not yet studied in the experimental research. This article reviews some health benefits of natural products that are more related to the prevention and treatment of muscle soreness.

**Saffron**

Saffron has been traditionally used in ancient medicine to cure various human diseases. It has many nonvolatile active components (Abdullaev, 2002 ▶), many of which are carotenoids, including zeaxanthin, lycopene, and various α- and β-carotenes. Recent experimental findings indicate that saffron's major compounds, crocin and crocetin, which are the derivatives of carotenoids, are powerful antioxidants (Asdaq and Inamdar, 2010 ▶), with anti-inflammatory (Poma et al., 2012 ▶) and antinociceptive (Hosseinzadeh and Shariaty, 2007 ▶; Hosseinzadeh and Younesi, 2002) activities. Recently, the oral supplementation of saffron (300 mg/day for 7 days before and 3 days after eccentric exercise) in a human study has shown a significant effect in terms of reducing DOMS symptoms (Meamarbashi and Rajabi, 2014 ▶). 

**Turmeric**

In the traditional medicine, turmeric is applied for reducing inflammation and pain. The most important chemical component of turmeric or curcuma Iongarhizomes is curcumin; tt has anti-inflammatory properties. In some experimental studies, curcumin has similar anti-inflammatory effects to some of the common nonsteroidal anti-inflammatory drugs (NSAIDs), like indomethacin, Vioxx, Celebrex, and ibuprofen. The molecular basis of the anti-inflammatory properties of curcumin is linked to its effects on several targets, including transcription factors, growth regulators, and cellular signaling molecules. Previous research has indicated its anti-inflammatory properties that reduce the activation of cyclooxygenase-2 (COX-2) and remove free radicals (Huang et al., 1991 ▶). Davis (2007) ▶ and others have provided the results of a decreased creatine kinase (CK) and inflammatory cytokine concentrations (Il-6, TNF-alpha, and IL-beta) in rats supplemented with curcumin compared with placebo. Curcumin ingestion has been reported to attenuate the cause of DOMS (Nicol et al., 2015 ▶; Tanabe et al., 2015 ▶). However, some research has indicated that curcumin has no effects on muscle soreness (Drobnic et al., 2014 ▶).

Curcumin is reported to directly influence the activity of various inflammatory regulators; it has been shown to reduce nuclear factor-kappaB (NF-KB) activation and activator protein 1 (AP-1) binding to DNA as well as to decrease the production of the COX-2 enzyme, all of which play a pivotal role in the inflammatory cascade (Chun et al., 2003 ▶; Han et al., 2002 ▶; Kang et al., 2004 ▶; Singh and Aggarwal, 1995 ▶). Furthermore, several studies have reported that curcumin can indirectly inhibit these inflammatory regulators through its ability to scavenge free radicals (Biswas et al., 2005 ▶; Rahman and Adcock, 2006 ▶). The effects of curcumin on the inflammation and recovery of running performance after downhill running in male mice has been reported and found to be effective in terms of reducing cytokines and creatine kinase enzyme (Davis et al., 2007 ▶).

**Caffeine**

Caffeine, also known as 1,3,7 trimethylxanthine, is a member of the family of methylated xanthine. Caffeine has been examined as a hypoalgesic. Reductions in ischemic forearm muscle pain have been found with the dose of 200 mg caffeine (Myers et al., 1997 ▶). Caffeine has also shown moderate to large hypoalgesic effects on quadriceps muscle pain during cycling exercise with the pre-exercise doses of 5 and 10 mg/kg, respectively (Motl et al., 2003 ▶; O'Connor and Cook, 1999 ▶). The most plausible mechanism of action for methylxanthines and its hypoalgesia is the competitive nonselective blockade of adenosine A_1_ and A_2_ receptors (Daly et al., 1999 ▶; James, 1997 ▶). Both caffeine and theophylline have been observed to have analgesic effects, while paraxanthine and theobromine do not have similar effects.

Caffeine implements its pharmacological effect primarily by blocking the adenosine A_1_, A_2A_, and A_2B_ receptors, but has less affinity for A_3_ receptors (Fredholm et al., 1999 ▶). These receptors are reported to be located in peripheral afferent nerves (Bryan and Marshall, 1999 ▶), the dorsal horn of the spinal cord, as well as higher brain areas associated with pain processing. Caffeine may inhibit phosphodiesterase, promote Ca^2+^ release, and block GABAA receptors if its consumption dose is one hundred times higher than its normal dietary use (Fredholm et al., 1999 ▶).

**Ginger**

Ginger (Zingiberofficinale) has analgesic and anti-inflammatory properties. There are many scientific pieces of evidence regarding the effectiveness of ginger for the alleviation of muscle soreness. Traditionally, ginger has been widely used for a variety of medicinal purposes, especially in the treatment of pain. One of the features of inflammation is increase in the oxygenation of arachidonic acid which is metabolized by two enzymatic pathways, cyclooxygenase (CO), and 5-lipoxygenase, leading to the production of prostaglandins and leukotrienes, respectively. Among the CO products, PGE_2_, and among the 5-lipoxygenase products, Leukotriene B4 (LTB_4_), are considered important mediators of inflammation. Ameliorative effects of ginger could be related to the inhibition of prostaglandin and leukotriene biosynthesis, i.e. it works as a dual inhibitor of eicosanoid biosynthesis (Srivastava and Mustafa, 1992 ▶). In a study about the effect of supplementation with ginger on muscle soreness, 74 adults who consumed ginger for 11 days had less muscle soreness after lifting weights. It is necessary to mention that the ingestion of single dose of 2 g ginger does not attenuate eccentric exercise-induced muscle pain, inflammation, or dysfunction 45 min after ingestion. However, ginger may attenuate the day-to-day progression of muscle pain (Black and O'Connor, 2010 ▶). Due to the paucity of well-conducted trials, the evidence for the efficacy of ginger for pain alleviation remains to be insufficient (Terry et al., 2011 ▶).

**Cinnamon**

Cinnamon or Cinnamomum zeylanicum has antioxidant and anti-inflammatory effects and its action is induced through decreasing the generation of reactive oxygen species (ROS) due to its phenolic and flavonoids contents in addition to modifying gene expression by inhibiting NF-kB activation (Azab et al., 2011 ▶). The obtained data suggest that cinnamon aqueous extract acts as a candidate radioprotector (Azab et al., 2011 ▶). Cinnamon has been used to treat dyspepsia, gastritis, blood circulation disturbance, and inflammatory diseases in many countries since the ancient age (Yu et al., 2007 ▶). The significant antiallergic, antiulcerogenic, antipyretic, anaesthetic, and analgesic activities have been verified by some researchers (Kurokawa et al., 1998 ▶). 

The anti-inflammatory mechanisms of Cinnamomum can be related to modulating macrophage-mediated inflammatory functions such as the over-production of cytokines, nitric oxide, and PGE_2_, adhesion molecule activation, as well as oxidative responses (Lee et al., 2006 ▶). 

Dietary ginger and cinnamon for reducing muscle soreness have been investigated in forty-nine female taekwondo players during the six week supplementation with 3 g dietary ginger and cinnamon, which showed significant changes in the muscle soreness, but not interleukin-6, in the cinnamon and ginger groups (Shokri Mashhadi et al., 2013 ▶). Oral consumption of 420 mg cinnamon per day, seven days before concentric exercise and three days after training, was effective for DOMS (Meamarbashi and Abbasian, 2013 ▶). This effect may be related to the effect of cinnamon on cell membrane integrity (Meamarbashi and Rajabi, 2013 ▶).

**Black tea**

Theaflavin and its derivatives are antioxidant polyphenols in tea leaves which are produced during the enzymatic oxidation (fermentation) of black tea. Consumption of theaflavin-enriched black tea extract could improve the recovery and reduce oxidative stress and DOMS responses to acute anaerobic intervals (Arent et al., 2010 ▶). 

**Pomegranate juice**

Pomegranate (Punicagranatum) contains anthocyanins, phytoestrogenic flavonoids and ellagic acid (Mousavinejad et al., 2009 ▶). Seventeen resistance trained men who were studied in a crossover design and supplemented with pomegranate juice showed a significant reduction in the elbow flexor muscles, but not knee extensor muscles (Trombold et al., 2011 ▶).

**Chamomile**

Chamomile (Matricariarecutita, Chamamelum mobile) is used for its sedative and antispasmodic, antiseptic, and anti-inflammatory effects. Chamomile contains important flavonoids, including apigenin, luteolin, and quercetin. Some alkylated flavonoids, such as chrysoplenin, chrysoplenol, and jaceidin, have been identified in it as well. In traditional medicine, chamomile is known to be a muscle relaxant; therefore, it may be helpful in reducing muscle soreness.

**Watermelon juice**

Watermelon (Citrulluslanatus) is a fruit rich of l-Citrulline (Tarazona-DÃaz et al., 2013 ▶). Its scientific name is 2-amino-5-(carbamoylamino) pentanoic acid. Citrulline is a precursor for arginine, which is involved in the formation of nitric oxide and creatine, and is a key constituent of the urea cycle, which detoxifies ammonia. Watermelon contains high concentration of lycopene, a carotenoid that may have beneficial effects on the risk of cancer and cardiovascular disease. Watermelon is also rich of vitamins A and C. Research on rats suggests that citrulline may reduce muscle fatigue (Goubel et al., 1997 ▶). It is believed that it can be used to reduce muscle soreness (Tarazona-DÃaz et al., 2013 ▶).

**Cherry juice**

In a research, the effectiveness of a fresh tart cherry juice for reducing the effects of eccentrically induced muscle damage was evaluated (Connolly et al., 2006 ▶). Tart cherries contain flavonoids and anthocynanins that have high antioxidant and anti-inflammatory properties that inhibiting the effects of cyclo-oxygenase (produce biological mediators) that cause inflammation and pain (Wang et al., 1999 ▶). 

**Garlic**

Garlic is used for the treatment of fatigue; however, its mechanism remains unclear (Morihara et al., 2007 ▶). The anti-fatigue function of garlic may be closely related to its many favorable biological and pharmacological effects. In animal studies, garlic has been shown to promote exercise endurance. In human studies, it has been confirmed that garlic produces symptomatic improvement in the persons with physical fatigue, systemic fatigue due to cold, or lassitude of indefinite cause, suggesting that garlic can resolve fatigue through a variety of actions. Currently, the available data strongly suggest that garlic may be a promising anti-fatigue agent and that further studies are required for elucidating its application are warranted (Morihara et al., 2007 ▶).

Allicin, a compound that results from crushing garlic, has been supplemented to reduce muscle damage resulting from eccentric exercise. Su et al. suggested that the anti-inflammatory and antioxidative capacities of allicin would decrease the inflammatory response and muscle damage after eccentric exercise (Su et al., 2008 ▶). Depending on its ingested form, garlic can have immune-enhancing or anti-oxidative capacities that typically result from many different biologically active compounds (Amagase et al., 2001 ▶). Effectiveness of garlic in muscle soreness can be related to its antioxidative properties. 

**Herb-drug interactions**

Herbal medicines are becoming increasingly popular. Even though in most countries, herbal medicines are being sold without prescription, medical guidance is necessary because of the adverse effects of these products and the potential for drug interactions. Some of the adverse effects and drug interactions reported for herbal products could be caused by impurities (e.g. allergens, pollen, and spores) (Hussain, 2011 ▶). 

Herbal supplements are often used in combination with the conventional drugs. Non-steroidal anti-inflammatory drugs (NSAIDs), particularly aspirin, have the potential to interact with herbal supplements with antiplatelet activity (ginkgo, garlic, ginger, bilberry, dong quai, feverfew, ginseng, turmeric, meadowsweet, and willow), with those containing coumarin (chamomile, motherworth, horse chestnut, fenugreek, and red clover), and with tamarind, enhancing the risk of bleeding (Abebe, 2002 ▶). The concomitant use of opioid analgesics with the sedative herbal supplements, chamomile, valerian, and kava may lead to increased central nervous system (CNS) depression (Abebe, 2002 ▶). Ginseng may also inhibit the analgesic effect of opioids. It is suggested that health-care professionals should be more aware of the potential adverse interactions between herbal supplements and analgesic drugs and take appropriate precautionary measures to avoid their possible occurrences. 

## Discussion

In the light of the available documents in relation to the effectiveness of natural products on DOMS, most researchers seem to favor the application of herbs in the prevention and treatment of DOMS. Currently, no intervention strategies exist for preventing DOMS, except evidence of preventive effect of 10 day supplementation with 300 mg saffron (Meamarbashi and Rajabi, 2014 ▶). Therefore, the most potent alternative is to treat the sign and symptoms when complaints occur. Due to the unknown pathology of DOMS, the unique alleviating method is not present. Hence, the effectiveness of therapeutic procedures is mostly dependent on the reduction degree of symptoms and signs and the duration of its effectiveness in combination with minimum possible side effects. 

People are increasingly seeking herbal remedies to relief pain, inflammation, and muscle soreness. Herbs are often believed to be “natural” and, therefore, safe; however, many mild to lethal side effects including toxic, allergic, interaction with drugs and other herbs have been reported (Bent and Ko, 2004 ▶). Herb-drug interaction often involves drug-metabolizing enzymes and drug transporter systems besides pharmacodynamic interaction (Hussain, 2011 ▶). In fact, the pharmacokinetic and pharmacodynamic characteristics of many herbs and commercial and natural dietary supplements are not completely recognized and potential interactions are not often foreseeable. Therefore, consumers should be aware of herbal side effects when frequently consuming herbs.

## Conflict of interest

There is no conflict of interest.
